# Case of Circulating Tumor Cells Discovered in Extensive Deep Venous Thrombosis in a Patient with Known Urothelial Carcinoma

**DOI:** 10.1155/2024/6144020

**Published:** 2024-03-09

**Authors:** Ekrem Yetiskul, Ali Kimyaghalam, Shahkar Khan, Yisroel Grabie, Taqi A. Rizvi, Salman Khan

**Affiliations:** Department of Internal Medicine, Staten Island University Hospital, 475 Seaview Avenue, Staten Island, NY 10305, USA

## Abstract

**Background:**

Currently, minimal data are available to explore the composition of venous thromboembolism in patients with cancer. This case report discusses a presentation of venous thromboembolism in a patient with high-grade urothelial carcinoma and highlights the pathology findings in thrombi. *Case Presentation*. A 55-year-old female who was diagnosed with high-grade urothelial carcinoma with multiple metastases developed an extensive deep vein thrombosis in her left lower extremity. Endovascular revascularization was indicated due to left lower extremity pain and swelling not responsive to anticoagulation. A mechanical thrombectomy was performed, and samples were sent for pathology. Pathologic examination discovered minute fragments of metastatic carcinoma, admixed with laminated blood clots (thrombus). The morphology of metastatic carcinoma and the immunostain profile were compatible with metastatic carcinoma of bladder origin.

**Conclusion:**

Cancer is a well-known risk factor for developing VTEs, and it is estimated that approximately 4–20% of cancer patients will experience VTE at some stage, the rate being the highest in the initial period following diagnosis. Annually, 0.5% of cancer patients will experience thrombosis compared with a 0.1% incidence rate in the general population (Elyamany et al., 2014). Despite knowing the increased incidence of VTEs in cancer patients, there are few studies to date that analyze the composition of thrombi in patients with cancer.

## 1. Introduction

Thrombosis is well-established as a common complication of malignancy and represents the second most frequent cause of death in cancer patients [1]. Cancer patients are generally hypercoagulable or prothrombotic, as they usually present with abnormalities in each component of Virchow's triad, thus contributing to thrombosis. The three components are a stasis of blood flow, endothelial injury, and hypercoagulability, the latter including abnormalities in the coagulation and fibrinolytic pathway and platelet activation. The specific mechanisms leading to abnormalities in Virchow's triad in cancer patients, particularly the effect on the host hemostatic system to promote the prothrombotic state, are not well understood and may be tumor-specific as different cancer types have varying risk rates for cancer-associated thrombosis. The data on the composition of the venous thrombi in patients with malignancies are lacking [2]. This case report discusses a presentation of venous thromboembolism in a patient with high-grade urothelial carcinoma and highlights the pathology findings present in thrombi.

## 2. Case Report

A 55-year-old female presented to the emergency room from a skilled nursing facility with a 1-day history of hematuria, left lower extremity swelling, and anemia with a hemoglobin level of 6.9. She endorsed developing multiple episodes of hematuria prior to presentation and noticed large blood clots in her urine. She also endorsed suprapubic pain, dysuria, urinary hesitancy, nausea, and one episode of non-bloody non-bilious emesis. Her medical history included recently diagnosed high-grade muscle-invasive urothelial bladder carcinoma with extensive metastasis. Her vital signs included a blood pressure of 192/103, pulse rate of 102, respiratory rate of 18, pulse oximetry of 97% on room air, and a temperature of 98.5 F. Pertinent findings on physical examination included suprapubic tenderness and 2+ pitting edema in bilateral lower extremities with tenderness to palpation and erythema in her left calf.

A complete blood count, comprehensive metabolic panel, coagulation studies, and urinalysis were performed on presentations, and results are presented in (Tables 1–4).

**Figure 1:**
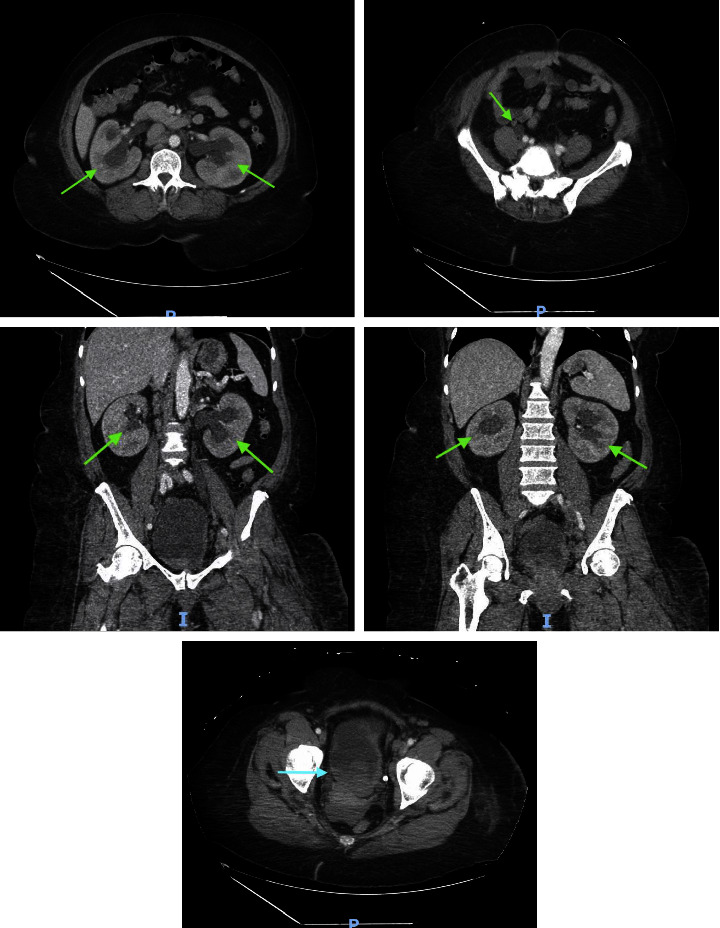
Axial and coronal views from computed tomography of the abdomen and pelvis with intravenous contrast demonstrating mild-to-moderate bilateral hydroureteronephrosis (green arrows) secondary to obstructing urinary bladder mass (blue arrow).

**Figure 2:**
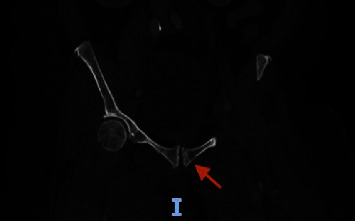
Coronal views from computed tomography of the abdomen and pelvis demonstrating left pubic lytic metastatic lesion (red arrow).

**Figure 3:**
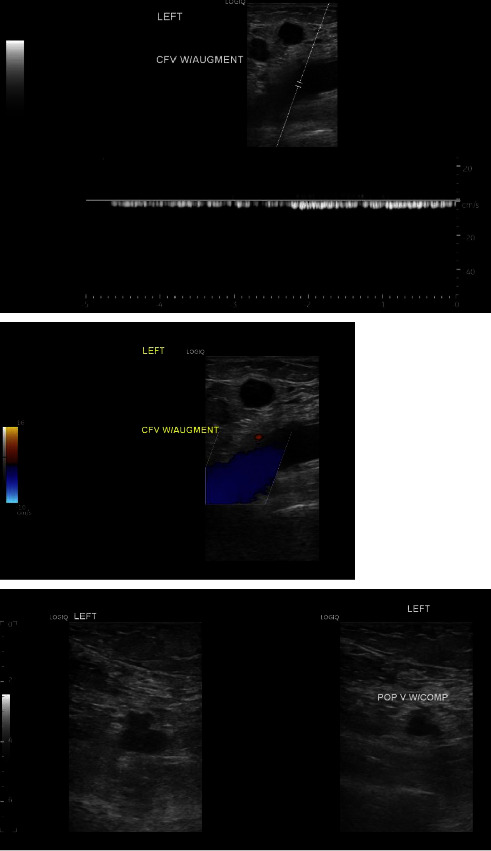
A bilateral lower extremity venous duplex ultrasound was performed on admission, which showed no evidence of deep venous thrombosis in either lower extremity.

A computed tomography of the abdomen and pelvis with intravenous contrast showed increased mild-to-moderate bilateral hydroureteronephrosis secondary to obstructing urinary bladder mass (Figure 1). There was also a new left pubic lytic metastatic lesion (Figure 2). A bilateral lower extremity venous duplex ultrasound was performed on admission, which showed severe edema in the left thigh with enlarged inguinal lymph nodes but no evidence of deep venous thrombosis in either lower extremity (Figure 3).

**Figure 4:**
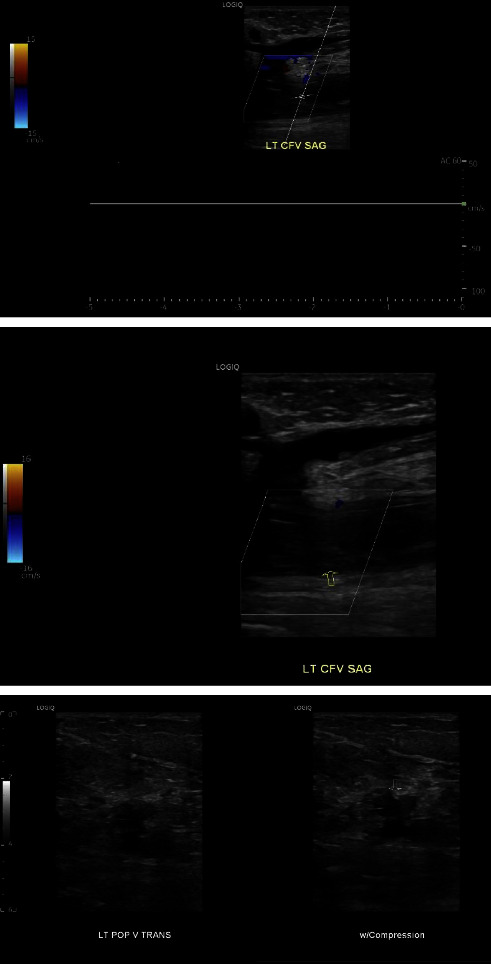
A repeat venous duplex was repeated during our patient's hospitalization which demonstrated deep vein thrombosis in the left common femoral vein, femoral vein, popliteal vein, gastrocnemius vein, peroneal vein, and posterior tibial vein. Demonstration of evidence of deep venous thrombosis in the left common femoral and left popliteal veins.

**Figure 5:**
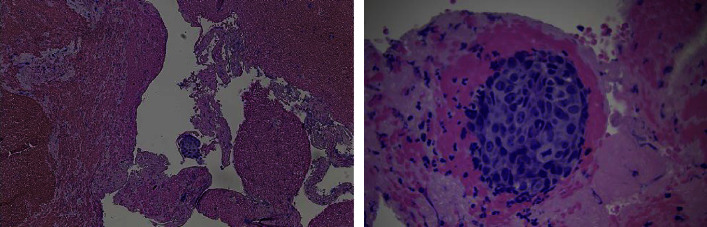
Pathologic examination of the left iliofemoral vein thrombus demonstrates minute fragments of metastatic carcinoma seen, admixed with laminated blood clots (thrombus).

The patient was admitted to the intensive care unit for severe hyponatremia, pyelonephritis, and AKI due to obstructive uropathy. She received hypertonic saline and was started on meropenem, given that she had a recent urine culture before the presentation, which was positive for extended-spectrum beta-lactamase (ESBL) *Escherichia coli* . Bilateral percutaneous nephrostomy catheters were placed on her second day of admission for obstructive uropathy. Palliative radiation therapy to her bladder was initiated early in her hospital admission. A total of 20Gy was given over 4 fractions and the patient tolerated treatment without difficulty. On hospital day eleven, a second bilateral lower extremity venous duplex ultrasound was performed for worsening left lower extremity pain, worsening edema, and erythema. The venous duplex demonstrated deep vein thrombosis in the left common femoral vein, femoral vein, popliteal vein, gastrocnemius vein, peroneal vein, and posterior tibial vein (Figure 4). There was also evidence of superficial thrombosis in the greater saphenous vein at the origin. No evidence of DVT or superficial thrombosis was noted in the right leg. The patient was not taking any anticoagulants prior to hospital presentation. Vascular surgery was consulted for recommendations regarding interventions, as the patient was having intermittent episodes of hematuria. A decision was made to start 1 mg/kg of enoxaparin twice daily with the possibility of performing a thrombectomy if the patient could not tolerate anticoagulation or if the patient's symptoms were not improving. Three days after starting anticoagulation, her lower extremity and pain did not improve, and a mechanical thrombectomy was performed. Samples of thrombi were sent for pathology by the vascular surgeon as the thrombus appeared abnormal with fragments of tan and pink soft tissue. Pathologic examination discovered minute fragments of metastatic carcinoma, admixed with laminated blood clots (thrombus) (Figure 5). The morphology of metastatic carcinoma and the immunostain profile were compatible with metastatic carcinoma of bladder origin. The patient's hospital course was further complicated by acute thrombocytopenia. She was diagnosed with heparin-induced thrombocytopenia, and fondaparinux was started for anticoagulation. The patient did not have recurring episodes of DVTs after the thrombectomy was performed.

## 3. Discussion

The occurrence of VTE in cancer patients remains an intricate and multifactorial scenario. Cancer has long been recognized as a potent risk factor for developing thromboembolism [3]. The dynamics of cancer-related hypercoagulability, often called Trousseau's syndrome, involves alterations in each component of Virchow's triad: endothelial dysfunction, stasis of blood flow, and a hypercoagulable state [1]. These mechanisms present an elevated risk of thrombosis in cancer patients. However, the intricacies of their interactions, especially the direct involvement of circulating tumor cells (CTCs) in promoting thrombosis, are an uncharted territory [2].

While the direct involvement of CTCs in thrombus formation remains a topic of study, it is hypothesized that CTCs can enhance thrombus formation both directly (becoming embolic or activation of platelets and coagulation cascade and indirectly (CTCs can secrete procoagulant substances, stimulate endothelial cells, or even produce microvesicles that promote thrombin generation) [4]. The principal mechanism by which malignancy induces a hypercoagulable state involves tissue factor (TF), a transmembrane glycoprotein that localizes coagulation factor VII/VIIa to the cell surface and activates the clotting cascade. TF is expressed on cancer cells as well as cancer cell-derived microparticles, which are vesicular structures released by multiple cell types and whose membranes retain the protein structures of their parent cells [5]. TF also aids in the survival of CTCs by preventing anchorage-dependent apoptosis (anoikis) via the PI3K/MAPK signaling pathways and by encouraging the epithelial-mesenchymal transition (EMT)—a transformative process where epithelial cells acquire characteristics of mesenchymal cells, including improved migratory capabilities [4]. In a study focusing on metastatic breast cancer (MBC), a filtration-based methodology was employed to enrich CTCs from the blood samples of patients [6]. This method successfully identified CTCs, with 97% (428 out of 442) demonstrating tissue factor expression [6]. Additionally, the research revealed through preclinical models that the epithelial-mesenchymal transition (EMT) leads to an increase in tissue factor expression in breast cancer cells [6]. Specifically, among the MBC CTCs that tested positive for the EMT marker vimentin, 89% (116 out of 130) also tested positive for tissue factor [6]. These findings indicate a potential mechanism through which tumor-derived and EMT-enhanced tissue factor expression by CTCs could contribute to systemic hypercoagulability [6]. A recent retrospective analysis by Gi et al. offers more profound insight into the presence of cancer cells within thrombi. They discovered the presence of cancer cells, either from direct vascular wall invasion or as small clusters, in thrombi in 27% of the analyzed VTE specimens associated with several different primary malignancies including lung, stomach, pancreatic and colorectal cancer [7]. This underscores the significant role of CTCs in the etiology of thromboembolic events in cancer patients.

Patients diagnosed with bladder cancer face a significant risk of experiencing thromboembolic events, affecting both veins and arteries [5]. Several extensive studies focused on large populations have documented a significantly elevated occurrence of venous thromboembolism (VTE) in individuals with bladder cancer [5]. In a study conducted by Blom et al. involving 2,250 patients, the frequency of VTE rose from 0.4 episodes per 100 patient-years in the 12 months leading up to the diagnosis to 1.3 episodes per 100 patient-years during the first six months following diagnosis [8]. This incidence reached its peak in patients suffering from metastatic disease, showing a rate of 3.1 episodes per 100 patient years [8]. Bladder cancer patients who undergo radical cystectomy are found to have TF-positive tumors and have a threefold higher risk of dying from their disease than TF-negative patients [9].

Furthermore, the treatment approach of VTE in cancer patients, especially in those with advanced stages of malignancies, is complex due to several factors, including the risk of bleeding associated with anticoagulation in the setting of thrombocytopenia or thrombus-associated cancer cells potentially being a nidus for metastasis [10]. Moreover, the presence of cancer cells within the thrombus, as highlighted by the pathology findings in this case, raises questions regarding the potential implications on VTE recurrence, metastasis, and the efficacy of anticoagulant therapy. While cancer-associated VTE's association with a hypercoagulable state is known, the direct implication of CTCs and cancer cells within thrombi suggests a more profound relationship between malignancy and thrombosis. Our case justifies the need for future research to better understand the full implications of these findings on improving the management and, therefore, the prognosis of cancer patients with VTE.

## 4. Conclusion

The nexus between VTE is well documented with cancer patients demonstrating a heightened risk for VTE, particularly in the initial phase following diagnosis. This underscores this association and highlights the presence of metastatic carcinoma fragments within the formed thrombus, affirming the notion of circulating tumor cells potentially playing a role in VTE formation. While the mechanistic link between cancer and thrombosis involves an interplay of factors, including surgery, chemotherapy-induced endothelial damage, stasis, and cancer-induced hypercoagulability, the detection of cancer cells within thrombi offers a tantalizing avenue for a deeper dive into the role of CTCs in VTE pathogenesis in the setting of malignancy. Clarifying the direct contribution of CTCs to VTE may allow us to discover potential avenues for tailoring thromboprophylaxis strategies, refining patient risk stratification, and improving patient outcomes overall [4].

## Figures and Tables

**Table 1:** Complete blood count on presentation.

White blood cell count	13.26 K/uL (4.80–10.80 K/uL)
Red blood cell count	3.01 M/uL (4.20–5.40 M/uL)
Hemoglobin	7.3 g/dL (12.0–16.0 g/dL)
Hematocrit	22.0% (37.0–47.0%)
Mean cell volume	73.1 fL (81.0–99.0 fL)
Mean cell hemoglobin concentration	33.2 g/dL (32.0–37.0 g/dL)
Red cell distribution width	13.6% (11.5–14.5%)
Platelet count	469 K/uL (130–400 K/uL)

**Table 2:** Comprehensive metabolic panel on presentation.

Sodium, serum	113 mmol/L (135–146 mmol/L)
Potassium, serum	5.0 mmol/L (3.5–5.0 mmol/L)
Chloride, serum	81 mmol/L (98–110 mmol/L)
Carbon dioxide, serum	16 mmol/L (17–32 mmol/L)
Anion gap, serum	16 mmol/L (7–14 mmol/L)
Blood urea nitrogen, serum	16 mg/dL (10–20 mg/dL)
Creatinine, serum	1.9 mg/dL (0.7–1.5 mg/dL)
Glucose, serum	129 mg/dL (70–99 mg/dL)
Calcium, total serum	8.4 mg/dL (8.4–10.5 mg/dL)
Protein total, serum	7.3 g/dL (6.0–8.0 g/dL)
Albumin, serum	3.1 g/dL (3.5–5.2 g/dL)
Bilirubin, total	0.3 mg/dL (0.2–1.2 mg/dL)
Alkaline phosphatase, serum	150 U/L (30–115 U/L)
Aspartate aminotransferase	23 U/L (0–41 U/L)
Alanine aminotransferase	10 U/L (0–41 U/L)
Estimated glomerular filtration rate	31 (≥60 mL/min/1.73 m^2^)

**Table 3:** Coagulation studies on presentation.

Prothrombin time, plasma	15.90 sec (9.95–12.87 sec)
INR	1.38 ratio (0.65–1.30 ratio)
Activated partial thromboplastin time	33.8 sec (27–39.2 sec)

**Table 4:** Urinalysis on presentation.

Color	Red (yellow)
Urine appearance	Turbid (clear)
Bilirubin	Moderate (negative)
Ketone -urine	Negative
Specific gravity	1.030 (1.008–1.030)
Protein, urine	100 mg/dL (negative)
Urobilinogen	<2 mg/dL (<2 mg/dL)
Nitrite	Positive (negative)
Leukocyte esterase concentration	Large (negative)
pH, urine	5.0 (5.0–8.0)
Blood, urine	Large (negative)
White blood cell, urine	15/HPF (0–5/HPF)
Red blood cell, urine	>720/HPF (0–4/HPF)
Bacteria	Moderate (negative)
Squamous epithelial cell	0 (0–5/HPF)
Glucose qualitative, urine	500 mg/d/L (negative)
